# Supplementary material for: Lifestyle behaviors and cardiovascular risk factors in transgender versus cisgender stroke survivors
Source: PLoS One. 2025 Sep 18;20(9):e0332206. doi: 10.1371/journal.pone.0332206 (PMC12445518; doi:10.1371/journal.pone.0332206)
Supplement: S1 Table — (DOCX) [file pone.0332206.s001.docx]

**S1 Table**

| **Variable Name** | **Label** | **Name Used in Article** |
| --- | --- | --- |
| _SEX | Calculated sex variable | Sex |
| _AGE_G | Imputed age in six groups | Age |
| _INCOMG1 | Computed income categories | Income Level |
| EDUCA | Education Level | Education Level |
| _RACEGR3 | Computed Five level race/ethnicity category | Race |
| _METSTAT | Metropolitan Status | Metropolitan Status |
| _STATE | State FIPS Code | Stroke Belt State Residency Status |
| RFSMOK3 | Current Smoking Calculated Variable | Tobacco Smoking |
| DRNKANY | Drink Any Alcoholic Beverages in Past 30 days | Alcohol Consumption |
| _RFBING5 | Binge Drinking Calculated Variable | Binge Drinking |
| ECIGNOW1 | Do you now use e-cigarettes, every day, some days, or not at all? | E-Cigarette Smoking |
| ADDEPEV3 | (Ever told) you had a depressive disorder | Depression |
| _MENT14D | Computed Mental Health Status | Poor Mental Health |
| BMI5CAT | Computed body mass index categories | Body Mass Index |
| TOTINDA | Leisure Time Physical Activity Calculated Variable | Low Physical Activity |
| DIABETE4 | (Ever told) you had diabetes | Diabetes |
| _RFHYPE6 | High Blood Pressure Calculated Variable | Hypertension |
| _RFCHOL3 | High Cholesterol Calculated Variable | High cholesterol |
| CVDCRHD4 | Ever Diagnosed with Angina or Coronary Heart Disease | Coronary Artery Disease |
| CVDSTRK3 | Ever Diagnosed with a Stroke | Stroke |
| TRNSGNDR | Do you consider yourself to be transgender? | Gender Identity |
