# Supplementary material for: Lifestyle behaviors and cardiovascular risk factors in transgender versus cisgender stroke survivors
Source: PLoS One. 2025 Sep 18;20(9):e0332206. doi: 10.1371/journal.pone.0332206 (PMC12445518; doi:10.1371/journal.pone.0332206)
Supplement: S2 Table — (DOCX) [file pone.0332206.s002.docx]

| **Risk Factors** | | **White (Unweighted percentage %)** | **Other Racial groups (Unweighted percentage %)** | **OR*** | **95% CI** | **P-value** |
| --- | --- | --- | --- | --- | --- | --- |
| **Tobacco Smoking** | | 17 (30.4%) | 30 (54.5%) | 0.363 | 0.167-0.791 | 0.013 |
| **Heavy Alcohol Consumption** | | 7 (12.5%) | 12 (23.5%) | 0.464 | 0167-1.291 | 0.205 |
| **Binge Drinking** | | 8 (14.3%) | 21 (39.6%) | 0.254 | 0.100-0.643 | 0.004 |
| **E-Cigarette Smoking** | | 3 (12.5%) | 10 (27.8%) | 0.371 | 0.090-1.525 | 0.210 |
| **Depression** | | 28 (47.5%) | 37 (62.7%) | 0.537 | 0.258-1.119 | 0.138 |
| **Poor Mental Health** | **0 days** | 29 (49.2%) | 21 (35.6%) | 0.645 | 0.420-0.989 | 0.045 |
|  | **1-13 days** | 16 (27.1%) | 13 (22.0%) |  |  |  |
|  | **14< days** | 14 (23.7%) | 25 (42.4%) |  |  |  |
| **Body Mass Index** | **Underweight** | 0 (0.0%) | 5 (11.1%) | 0.951 | 0.616-1.466 | 0.818 |
|  | **Normal** | 12 (23.1%) | 8 (17.8%) |  |  |  |
|  | **Overweight** | 21 (40.4%) | 6 (13.3%) |  |  |  |
|  | **Obese** | 19 (36.5%) | 26 (57.8%) |  |  |  |
| **Low Physical Activity** | | 20 (33.3%) | 22 (37.9%) | 0.818 | 0.385-1.740 | 0.701 |
| **Diabetes** | | 17.3 (28.3%) | 31 (52.5%( | 0.357 | 0.167-0.763 | 0.009 |
| **Hypertension** | | 14 (53.8%) | 23 (60.5%) | 0.761 | 0.277-2.086 | 0.616 |
| **Hypercholesterolemia** | | 14 (60.9%) | 12 (44.4%) | 1.944 | 0.628-6.021 | 0.272 |
| **Coronary Artery Disease** | | 18 (31%) | 25 (43.1%) | 0.594 | 0.277-1.272 | 0.249 |

*The reference group for the odds ratios is “other racial groups”
