# Supplementary material for: Lifestyle behaviors and cardiovascular risk factors in transgender versus cisgender stroke survivors
Source: PLoS One. 2025 Sep 18;20(9):e0332206. doi: 10.1371/journal.pone.0332206 (PMC12445518; doi:10.1371/journal.pone.0332206)
Supplement: Attachment — Submitted filename: review notes PLOS.docx [file pone.0332206.s003.docx]

**Review notes for author**

- Line 1 on measurement a coma is missing after birth.
- The measurement of mental health status; what are the three mental health groups, and which one defines suboptimal mental health or poor mental health.
- Where is the figure 1 and 2 mentioned in the manuscript they are nowhere to be seen.

**Results section:**

- what is 25k this is not standard is it dollars, pounds?
- Table structure needs to be standardized, upper boarder lines missing, edit the tables to standard publishable table formats.
- On 3.2; author should tell us which lifestyle risk factor had higher odds, and when author uses rates, it does not line up with cross-sectional nested case control study. Better to stick with odds ratio. When author says rates it as if the lifestyle risk factors were measured as incident risks and yet this is a snapshot at a time no follow us to talk about incidence rates.
- Table column tittles should also indicate n in addition to %

**Discussion section.**

- The source of the statement “recent studies show 1-6%rate of stroke should be referenced.
- Author should use prevalence instead of rate which is appropriate for this cross-sectional study. The author talks about stroke prevalence and then discusses rate it should be in harmony.
- The study examined post stroke risk factors its not clear when stroke was diagnosed but it’s understandable since it’s a cross-sectional study, but these post stroke risk factors were they assessed as preexisting or occurring after the stroke had started in the telephone calls? it would do better to clarify this. Did they develop because of transgender status or even before that status. The transgender is mentioned as different from sex assigned at birth, so is there a timeline for these risk factors in this population.
- Binge drinking is not discussed and yet it has a significant association with post stroke survivors in transgender according to the results. why? How does its risk differ from alcohol drinking as a maladaptive strategy in this population group.
- Discussion also talks about smoking, is this the same as tobacco smoking that had significant odds. Smoking may be of other substances like non-tobacco, the terms need to be consistent.
- Bias may not only be due to stigma but also recall bias and classification especially on some factors like binge drinking, duration and diagnosis of stroke. This weakness needs to be well elaborated.
- The study investigated post stroke risk factors and not outcomes. Outcomes of stroke would include hospitalization, worsening stroke or death. But the study focused on risk factors and that is what the statements should reflect.

**Conclusion section**

- Conclusion should focus on study outcomes, **post** stroke risk factors.
- There is no need of bringing hormonal therapy in the conclusion when you have not studied it and it is nowhere in the results.it can stay in discussion.
- Author talks about enhancing post stroke outcomes in the last paragraph; is it good outcomes being talked about here. The conclusion should focus on recommendations for risk reduction for post stroke transgender individuals.
