# Supplementary material for: Lifestyle behaviors and cardiovascular risk factors in transgender versus cisgender stroke survivors
Source: PLoS One. 2025 Sep 18;20(9):e0332206. doi: 10.1371/journal.pone.0332206 (PMC12445518; doi:10.1371/journal.pone.0332206)
Supplement: Attachment — Submitted filename: Reviewers Response.docx [file pone.0332206.s005.docx]

#Editor

1. Please ensure that your manuscript meets PLOS ONE's style requirements, including those for file naming. The PLOS ONE style templates can be found at

<https://journals.plos.org/plosone/s/file?id=wjVg/PLOSOne_formatting_sample_main_body.pdf> and

[https://journals.plos.org/plosone/s/file?id=ba62/PLOSOne_formatting_sample_title_authors_affiliations.pdf](https://track.editorialmanager.com/CL0/https:%2F%2Fjournals.plos.org%2Fplosone%2Fs%2Ffile%3Fid=ba62%2FPLOSOne_formatting_sample_title_authors_affiliations.pdf/1/010f0196f96b3426-e6d74494-7c31-48b8-bcdf-2b3583002410-000000/WAx_eSNfKbI_xnXBB0G27eCej06itqTGbggfWHauvdM=212)

Thank you for your valuable comments. The journal's style requirements were reviewed, and corresponding adjustments were made to the manuscript.

2. Your ethics statement should only appear in the Methods section of your manuscript. If your ethics statement is written in any section besides the Methods, please move it to the Methods section and delete it from any other section. Please ensure that your ethics statement is included in your manuscript, as the ethics statement entered into the online submission form will not be published alongside your manuscript.

Thanks for the comment. An ethics statement is included in the manuscript's methods section.

3. Please include a separate caption for each figure in your manuscript.

Thank you. Your recommendation has been applied.

4. Please review your reference list to ensure that it is complete and correct. If you have cited papers that have been retracted, please include the rationale for doing so in the manuscript text, or remove these references and replace them with relevant current references. Any changes to the reference list should be mentioned in the rebuttal letter that accompanies your revised manuscript. If you need to cite a retracted article, indicate the article’s retracted status in the References list and also include a citation and full reference for the retraction notice.

Thank you for the comment. No retracted articles are cited in the manuscript. References 35,36,40,41,51-56 have been added to the manuscript.

\# Reviewer 1

Issue: The study does not include data on hormone therapy, which is a significant factor influencing stroke risk in transgender individuals.
Suggestion: Acknowledge this limitation more explicitly in the discussion and suggest future studies to investigate the role of hormone therapy in stroke risk.

Thank you for your valuable comments. The discussion on hormone therapy has been expanded as you recommended.

Limited Data on Healthcare Access:
Issue: The manuscript does not explore disparities in healthcare access, which could influence post-stroke outcomes.
Suggestion: Include a discussion on how healthcare access barriers may exacerbate stroke risk and outcomes in transgender individuals.

Thank you for the comment. The recommended section has been added to the manuscript discussion per the information you provided.

Mental Health Analysis:
Issue: While trends in depression and poor mental health are noted, they are not statistically significant.

Suggestion: Consider exploring qualitative data or longitudinal studies to better understand the relationship between mental health and stroke risk in transgender populations.

Thank you for your comment. A paragraph about depression and poor mental health has been added to the manuscript.

Classification Bias:

Issue: Stroke diagnoses in the BRFSS dataset are self-reported, which may introduce classification bias.

Suggestion: Discuss how this limitation could impact the findings and suggest validation through clinical data in future research.

Thanks for the comment. The discussion did mention classification bias, but we expanded on it in more detail.

Sample Size:

Issue: The sample size for transgender stroke survivors is relatively small (n=189), which may limit the generalizability of the findings.
Suggestion: Highlight the need for larger, more representative studies to confirm these results.

Thank you. We have added this point to the limitations.

Discussion of E-Cigarette Use:

Issue: The manuscript notes a significant disparity in e-cigarette use but does not delve into its implications for stroke risk.
Suggestion: Expand the discussion on the cardiovascular risks associated with e-cigarette use and its potential role in stroke prevention strategies.

Thank you very much. We have discussed this issue in greater detail.

Figures and Tables:

Issue: Some tables (e.g., Table 3) are dense and difficult to interpret.
Suggestion: Simplify the presentation of key findings by summarizing them in a more reader-friendly format, such as bullet points or visual aids.

Thank you for your comment. It appears that presenting the table in a standardized format has resolved the issue with its interpretation. The highlights also briefly summarize the key findings.

Future Directions:

Issue: The manuscript does not provide specific recommendations for future research.
Suggestion: Include a dedicated section outlining areas for further investigation, such as the impact of hormone therapy, healthcare access, and longitudinal studies on post-stroke outcomes.

Thanks for the comment. The recommended section has been added at the end of the discussion.

\# Reviewer 2

As authors mentioned in the discussion, the results of this study describes the comparison of characteristics of post-stroke residents in two groups. so using "stroke risk factor" phrase is not applicable for topic. They search DM, HT, HL, behavioral risk factors but, it i not evident that these factors were present before stroke. So I suggest to change the Title "Assessing stroke risk disparities" as it sounds that you designed the study to find out the preexisting risk factors for stroke in transgender groups.

Thank you for your valuable comments. We have revised the manuscript's subject title to **"Post-Stroke Health Disparities:** Comparative Analysis of Lifestyle and Vascular Profiles in Transgender and Cisgender Stroke Survivors" to address and resolve this issue.

There are some punctuation and font size discordance, please check for them.

Thank you for your valuable comment. There were some errors in the text, which have been corrected.

Please check for reference style, and correct.

The manuscript has been reviewed for referencing style and corrected.

#Reviewer 3

Line 1 on measurement a coma is missing after birth.

Thank you for your valuable comments. Our intended meaning was **birth sex**, and to convey the concept more precisely, we changed it to **assigned sex at birth**.

The measurement of mental health status; what are the three mental health groups, and which one defines suboptimal mental health or poor mental health.

Thank you for your comment. The definition of each group has been added to the Measurements section.

Where is the figure 1 and 2 mentioned in the manuscript they are nowhere to be seen.

Thanks for the comment. The figure captions have been incorporated into the manuscript file, and the figures have also been provided separately.

**Results section:**

what is 25k this is not standard is it dollars, pounds?

Thank you for the comment. The amounts are in US dollars, as indicated by the dollar sign placed next to them.

Table structure needs to be standardized, upper boarder lines missing, edit the tables to standard publishable table formats.

Thanks for your comment. The tables have been modified to an standard and more understandable form.

On 3.2; author should tell us which lifestyle risk factor had higher odds, and when author uses rates, it does not line up with cross-sectional nested case control study. Better to stick with odds ratio. When author says rates it as if the lifestyle risk factors were measured as incident risks and yet this is a snapshot at a time no follow us to talk about incidence rates.

Thank you for the comment. You are right; in this section, the word "rate" has been replaced with "odds" to be more appropriate.

Table column tittles should also indicate n in addition to %

Thank you. The table column titles have been modified per your recommendation.

**Discussion section.**

The source of the statement “recent studies show 1-6%rate of stroke should be referenced.

Thanks for the comment. The referencing has been corrected.

Author should use prevalence instead of rate which is appropriate for this cross-sectional study. The author talks about stroke prevalence and then discusses rate it should be in harmony.

Thank you for your comment. As per your recommendation, the word rate has been changed to prevalence.

The study examined post stroke risk factors its not clear when stroke was diagnosed but it’s understandable since it’s a cross-sectional study, but these post stroke risk factors were they assessed as preexisting or occurring after the stroke had started in the telephone calls? it would do better to clarify this. Did they develop because of transgender status or even before that status. The transgender is mentioned as different from sex assigned at birth, so is there a timeline for these risk factors in this population.

Thank you for the comment. Because BRFSS data are collected via telephone interviews, the temporal sequence of stroke and associated risk factors remains unclear. It is not possible to determine whether these factors developed prior to stroke onset or emerged afterward. Similarly, the timing of gender identity development relative to stroke and risk factor accumulation cannot be established from cross-sectional data. This point has also been added to the discussion.

Binge drinking is not discussed and yet it has a significant association with post stroke survivors in transgender according to the results. why? How does its risk differ from alcohol drinking as a maladaptive strategy in this population group.

Thank you very much. We intended to categorize binge drinking as a subset of alcohol drinking in this section; however, based on your recommendation, it was more appropriate to consider the two separately, and we have implemented this change.

Discussion also talks about smoking, is this the same as tobacco smoking that had significant odds. Smoking may be of other substances like non-tobacco, the terms need to be consistent.

Thank you for the comment. The intended meaning was specifically tobacco smoking, and we replaced the word smoking with tobacco smoking in the text to enhance clarity.

Bias may not only be due to stigma but also recall bias and classification especially on some factors like binge drinking, duration and diagnosis of stroke. This weakness needs to be well elaborated.

Thank you for your comment. The discussion of biases in the limitations section has been expanded.

The study investigated post stroke risk factors and not outcomes. Outcomes of stroke would include hospitalization, worsening stroke or death. But the study focused on risk factors and that is what the statements should reflect.

**Conclusion section**

Conclusion should focus on study outcomes, **post** stroke risk factors.

Thank you. Yes, using post-stroke risk factor conveys the intended meaning more clearly, and we have made this change.

There is no need of bringing hormonal therapy in the conclusion when you have not studied it and it is nowhere in the results.it can stay in discussion.

Thanks for your comment. This part has been removed from the conclusion.

Author talks about enhancing post stroke outcomes in the last paragraph; is it good outcomes being talked about here. The conclusion should focus on recommendations for risk reduction for post stroke transgender individuals.

Thanks for the comment. Some recommendations for risk reduction for stroke survivor transgenders have been added.
