# Supplementary material for: Lifestyle behaviors and cardiovascular risk factors in transgender versus cisgender stroke survivors
Source: PLoS One. 2025 Sep 18;20(9):e0332206. doi: 10.1371/journal.pone.0332206 (PMC12445518; doi:10.1371/journal.pone.0332206)
Supplement: Attachment — Submitted filename: Editor response trans.docx [file pone.0332206.s006.docx]

Response to the editor

Title:
This has several issues.
What is meant by “vascular profiles”? It is vague and misleading. The study examines specific cardiovascular risk factors (CAD, HTN, DM, HLD) and not comprehensive vascular profiles, which would include imaging data, biomarkers or physiological measurements none of which was included.
Using disparities in the title and eliminating race and income level from your matched analysis is problematic as these are known drivers of healthcare disparities.
The title does not clarify that the study is about risk factors present in stroke survivors and not necessarily factors that developed after stroke or preceded stroke, which a cross-sectional design cannot determine.
I suggest an alternative title:
Lifestyle behaviors and cardiovascular risk factors in transgender versus cisgender stroke survivors. Thank you for your valuable comments. We have revised the title accordingly, as suggested.

Abstract:
The matching was 189 transgender to 378 cisgender (from the methods section). The total sample size stated in the abstract can be misleading, as you only analyzed 567 individuals. Please edit this to avoid confusion. Thank you for pointing out this important clarification. the abstract has been corrected to indicate that 189 transgender stroke survivors were matched with 378 cisgender stroke survivors.

Highlights:
• The statement that transgender individuals showed 70% higher prevalence of stroke is misleading. Please interpret odds ratios appropriately. Thank you for pointing this out. We have corrected the statement as recommended.

• Smoking and alcohol: Please do not conflate likelihood and odds ratio. Again, interpret this properly. Thank you for your comment. There were errors in the reported odds ratios, which were identified and corrected according to your recommendations.

• E-cigarette: I suggest a review of the proper interpretation of odds ratios and proper reporting here.
Thanks for your comment. The issues related to the interpretation of the odds ratios have been resolved.

Introduction:
This section needs to be improved. Are there putative mechanisms (beyond hormone therapy) that adversely increase the risk of strokes in transgender patients. This may be obvious to the authors but must be clearly stated as it forms that bases of the scientific value of your investigations. A focused survey of the current literature on disparities in risk factors and cardiovascular disease outcomes is needed. Thank you for this suggestion. The Introduction has been expanded to outline additional mechanisms beyond hormone therapy, including traditional vascular risk factors, psychosocial determinants, and healthcare access barriers.

Please frame the distinction between pre-stroke risk factors and post-stroke characteristics. Thank you for the comment. We have added the distinction between pre-stroke risk factors and post-stroke characteristics to the manuscript.

Methods:
Study population: Were nonbinary individuals classified as cisgender or transgender?
Thanks for your comment. Nonbinary individuals were classified as transgender, as mentioned in the text.

“Their birth gender determined the gender of respondents”: Please edit it for clarity. Thank you. We revised this for clarity, now stating explicitly that respondents’ gender was identified based on their assigned sex at birth, consistent with BRFSS definitions.

Please report on the BMI cut-offs for the nominal scale introduced. We have added the BMI cut-off points (underweight <18.5, normal 18.5–25, overweight 25–30, obese ≥30) in the Methods section.

Statistical analysis: Thank you for including information on why race and income level were not included in the list of variables on which your participants were matched. However, this justification is problematic, and this approach may introduce substantial bias and limits the representativeness of findings. If you can provide evidence from literature, especially from studies that use the BRFSS, your justification may be strengthened.
Thank you for this point. We cited BRFSS-based literature (e.g., Pierannunzi et al.) supporting this methodological choice.

Results:
There are several instances where the authors conflate odds ratios with likelihood. This needs to be corrected. Thanks for the comment. This issue, which was present in certain parts of the manuscript, has now been resolved.

Some adjusted ORs show dramatic changes from crude ORs, suggesting potential overadjustment or multicollinearity. Please consider pre-estimation multicollinearity checks such as correlation matrix or post-estimation checks such as VIF to inform which variables you include in your final model.
Thank you for your comment. We had considered the possibility of multicollinearity and overadjustment. The variables included in the final model were selected based on both clinical relevance and statistical considerations, and no concerning multicollinearity was observed. Therefore, the adjusted ORs can be considered robust.

Table 1: Sex (please show that this is as assigned at birth)
Thank you. Your recommendation has been applied.

Table 2:
• Please state currency for income level. Are these median household incomes or unadjusted reports by the respondents? State clearly that these are US Dollars. Thank you for your comment. The income levels reported in our study are unadjusted self-reports by the respondents, and we have clarified in the manuscript that the values are expressed in US Dollars.

• Please explain why you include this table and caption indicating PS matching when the methods say the variables were excluded from PS matching. The second table refers to the demographic variables that were not included in the matching. After performing PSM, these variables have been compared between the two groups. For better clarity, we have provided an explanation within the text.

Table 3:
No correction for multiple testing despite numerous comparisons increasing Type I error risk. Please apply appropriate multiple comparison corrections or acknowledge this limitation and interpret p-values more cautiously.
Thanks for this comment. We have acknowledged the increased risk of Type I error due to multiple comparisons in the limitations and noted that p-values should be interpreted cautiously.

Lifestyle Risk Factors:
Did you check for collinearity. Binge drinking and alcohol consumption appear to be collinear intuitively. Including both in your models may be concerning on the surface. Please provide post estimation tests such as VIF.
We appreciate your insightful comment. We would like to clarify that in our adjusted logistic regression models, only alcohol consumption was included as a confounding variable, while binge drinking was not part of the adjustment set.

Discussion and Conclusions:
The 3rd sentence of the second paragraph is misleading. Thank you. This sentence has been revised.

I advise caution in interpreting the findings of the small sample cross-sectional study. Avoid language that suggests causal implications. Thank you for your advice. We have revised the text to avoid causal wordings and kept the interpretation consistent with the cross-sectional design.

Please discuss why aORs differ so much from cORs. Thank you. We added an explanation that the differences between cORs and aORs reflect confounding variables, highlighting the importance of multivariable analysis.

Please explore alternative explanations for your findings. Thanks for the comment. We have included alternative explanations for the findings as much as possible in the Discussion section.

Please acknowledge that self-reported diagnosis without independent validation introduces potential bias, particularly for stigmatized factors. Discuss this as a limitation. Thank you for the comment. We acknowledged this as a limitation and noted the potential for misclassification bias due to reliance on self-reported diagnoses and stigmatized conditions.

Discuss potential survivor bias. Thank you for your comment. This limitation has been addressed in the Discussion.

Paragraph 11: The study did not study post-stroke outcomes. Please correct this. Thanks for your comment. The mentioned part has been revised.

Final comment:
Despite the limitations above and those raised by the external reviewers, the study provides valuable preliminary data on this underserved population. The manuscript STILL REQUIRES MAJOR revisions before publication. The most critical issues are misinterpretation of statistical measures and overstatement of causal implications. With appropriate careful revisions, this could be a valuable contribution to our understanding of health disparities in transgender populations.
Thank you for all the comments provided to improve the manuscript. We have attempted to address all interpretive, structural, and punctuation issues and have revised the manuscript’s shortcomings according to your recommendations. We hope that the manuscript will meet the standards of your esteemed journal.
